# A Computational Approach to Finding Novel Targets for Existing Drugs

**DOI:** 10.1371/journal.pcbi.1002139

**Published:** 2011-09-01

**Authors:** Yvonne Y. Li, Jianghong An, Steven J. M. Jones

**Affiliations:** Canada's Michael Smith Genome Sciences Centre, British Columbia Cancer Agency, Vancouver, British Columbia, Canada; University of California San Diego, United States of America

## Abstract

Repositioning existing drugs for new therapeutic uses is an efficient approach to drug discovery. We have developed a computational drug repositioning pipeline to perform large-scale molecular docking of small molecule drugs against protein drug targets, in order to map the drug-target interaction space and find novel interactions. Our method emphasizes removing false positive interaction predictions using criteria from known interaction docking, consensus scoring, and specificity. In all, our database contains 252 human protein drug targets that we classify as reliable-for-docking as well as 4621 approved and experimental small molecule drugs from DrugBank. These were cross-docked, then filtered through stringent scoring criteria to select top drug-target interactions. In particular, we used MAPK14 and the kinase inhibitor BIM-8 as examples where our stringent thresholds enriched the predicted drug-target interactions with known interactions up to 20 times compared to standard score thresholds. We validated nilotinib as a potent MAPK14 inhibitor *in vitro* (IC50 40 nM), suggesting a potential use for this drug in treating inflammatory diseases. The published literature indicated experimental evidence for 31 of the top predicted interactions, highlighting the promising nature of our approach. Novel interactions discovered may lead to the drug being repositioned as a therapeutic treatment for its off-target's associated disease, added insight into the drug's mechanism of action, and added insight into the drug's side effects.

## Author Summary

Most drugs are designed to bind to and inhibit the function of a disease target protein. However, drugs are often able to bind to ‘off-target’ proteins due to similarities in the protein binding sites. If an off-target is known to be involved in another disease, then the drug has potential to treat the second disease. This repositioning strategy is an alternate and efficient approach to drug discovery, as the clinical and toxicity histories of existing drugs can greatly reduce drug development cost and time. We present here a large-scale computational approach that simulates three-dimensional binding between existing drugs and target proteins to predict novel drug-target interactions. Our method focuses on removing false predictions, using annotated ‘known’ interactions, scoring and ranking thresholds. 31 of our top novel drug-target predictions were validated through literature search, and demonstrated the utility of our method. We were also able to identify the cancer drug nilotinib as a potent inhibitor of MAPK14, a target in inflammatory diseases, which suggests a potential use for the drug in treating rheumatoid arthritis.

## Introduction

The continuing decline of drug discovery productivity has been documented by many studies. In 2006, only 22 new molecular entities were approved by the Food and Drug Administration (FDA) despite research and development expenditures of $93 billion USD by biotech companies and large pharmaceutical companies, and this low productivity has not improved since [1]. From discovering, developing to bringing one new drug to market, clinical trials are the most expensive step, accounting for 63% of the overall cost [2]. To this end, drug repositioning - finding new therapeutic indications for existing drugs - represents an efficient parallel approach to drug discovery, as existing drugs already have extensive clinical history and toxicology information.

Many of today's repositioned drugs were discovered through serendipitous observations, including high profile drugs sildenafil by Pfizer - first developed for angina but later approved for erectile dysfunction - and thalidomide by Celgene - first marketed for morning sickness, then approved for leprosy and recently for multiple myeloma [3]. Repositioned drugs have also been discovered through rational observations, including imatinib (Gleevec), which was first approved for chronic myeloid leukemia by targeting the BCR-Abl fusion protein but was subsequently approved for gastrointestinal stromal tumor due to its ability to potently inhibit c-KIT [4]. Another example is the anti-depressant duloxetine (Cymbalta) that is also indicated for stress urinary incontinence based on a shared mechanism of action between the two diseases [3]. In order to rationally reposition drugs, novel target-disease or drug-target relationships must first be elucidated.

By screening compounds against a panel of proteins, there is potential to discover novel drug-target interactions. Drug candidates are routinely screened against a small panel of similar proteins to determine their specificity to the intended target. Large panels with hundreds of kinase proteins have been developed to assess kinase inhibitor specificity [5], especially since we now know that many kinase drugs are multi-targeting. However, the druggable proteome is much larger than just the kinome, so larger and more varied protein panels are needed to truly assess drug specificity. With the availability of massively parallel DNA sequencing technology, recurrently mutated proteins in diseases – such as EZH2 in certain lymphomas [6] and FOXL2 in certain ovarian cancers [7] - are now being rapidly determined and are also relevant drug targets. However, testing all drugs against all targets experimentally is extremely costly and technically infeasible.

Recent computational endeavors to predict novel drug repositioning candidates have used methods incorporating protein structural similarity [8], chemical similarity [9], or side effect similarity [10]. One study also incorporated some molecular docking to help filter interactions predicted through protein binding site similarity [8]. Here we present a large-scale molecular docking analysis of known drugs against known protein targets for the prediction of novel drug-target interactions. Molecular docking is a computational method that predicts how two molecules interact with each other in 3-dimensional space. It is well established as a virtual screening method in drug discovery [11], where typically many chemicals are docked against a specific protein binding site, in order to discover novel inhibitors of that target. Compared to similarity analyses, docking has the potential to find drugs that bind to proteins with novel scaffolds as well as off-targets that may be structurally dissimilar to the known targets.

Large-scale docking of many targets to many drugs is now feasible when run on powerful computer clusters. However, limitations in scoring methods result in high false positive prediction rates [12], and large-scale studies amplify these low prediction accuracies. Our method emphasizes removing false positive predictions using scoring and ranking thresholds, and retaining only the highest confidence interactions as drug repositioning candidates.

## Results

### Computational pipeline

**Figure 1:**
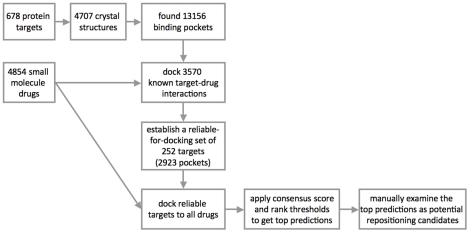
The computational molecular-docking pipeline.

A computational pipeline was developed for large-scale molecular docking of drugs to protein targets (Figure 1). Briefly, we collected all 3D structures available for each drug target, determined binding pockets in the structures, and docked drugs to each pocket. Results were collected and thresholds were applied to select the top predicted interactions, which were then visually inspected.

### Known interactions docking

We first docked 3570 known protein-drug interactions annotated by DrugBank, between 678 unique human proteins and 1309 small molecule drugs. We used the docking software ICM developed by Molsoft [13], which ranks ligands using a Monte-Carlo based docking procedure and an empirical, energetics-based docking score. Like most docking software, ICM recommends a standard score cut-off for virtual screening efforts: −32 [14], where more negative scores represent more likely binding interactions. However, studies have used different cut-offs depending on the protein target [15]. Here we used a score of −30 as the threshold for ‘good’ dockings scores. Of the 3570 known interactions docked, 1116 (31%) had a good ICM docking score. 252 proteins had at least one known interaction predicted by docking – these formed the ‘reliable’ set of proteins that we believe are more suited for docking purposes. A breakdown of protein classifications for this reliable set revealed that 67% of targets were enzymes, of which 12% were protein kinases. In contrast, there were few G-protein coupled receptors in our database due to lack of crystal structures, which reflects both the current state of solved protein crystal structure space as well as popular drug targets.

### Known interactions docking evaluation

In high-throughput molecular docking, it is common to hold protein structures rigid during the simulation. With this restriction, re-docking a PDB ligand back to its native PDB structure (cognate docking) is a simpler task than docking a different ligand to the structure (non-cognate docking) because in the former case the protein is already in a specific ligand-bound conformation. Cognate-docking situations occur frequently and previous studies show that they can be docked well in 60–80% of cases [16]. In contrast, the more useful non-cognate docking is only successful in 20–40% of cases [16].

**Figure 2:**
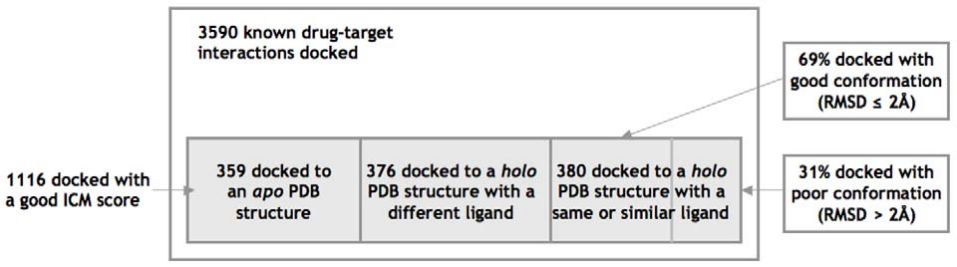
Evaluating the known drug-target docking. 1116 (31%) of 3570 known interactions docked with a good score. Two-thirds of the 1116 were ligands docking to non-cognate protein structures, showing that the method could do more than re-dock existing drug-target structures.

We analyzed the 1116 known interactions to examine whether those that docked well were only due docking cognate ligands. For each interaction, we observed whether the drug bound 1) a *holo* (unliganded) protein structure, 2) an *apo* (liganded) structure with a same or similar ligand as the drug (the cognate-docking scenario), or 3) an *apo* structure with a chemically different ligand from the drug. Chemical similarity was defined as having a Tanimoto coefficient less than 0.54. Figure 2 shows that cognate docking occurred in 380 of the 1116 interactions. Of these, only 56 were drugs docked to an *apo* protein with the same ligand (Tanimoto coefficient of 0). The majority of drugs docked well to *holo* structures as well as *apo* structures with dissimilar ligands. In short, the ICM docking method was able to predict known interactions for both cognate and non-cognate docking scenarios.

Aside from the docking score, it was also important to verify that the ligands were docked in correct binding conformations. We further examined the 380 cognate dockings and found that the docked drug conformation was close to the known drug conformation (RMSD value ≤2 Å) in 69% of cases. The other 31% fell into two categories: 1) partly symmetrical ligands like NAD and 2) ligands that bound to a small pocket. In the first case, the molecule was incorrectly determined to be flipped, causing a high RMSD; however, its central portion was docked correctly due to symmetry. In the second case, the region of ligand bound in the pocket was docked correctly, but the region free in solvent contributed to a poor RMSD value. Overall, this analysis showed that when a known interaction was docked with a good score, the binding conformation was also reasonably predicted.

### Known drug-target network

**Figure 3:**
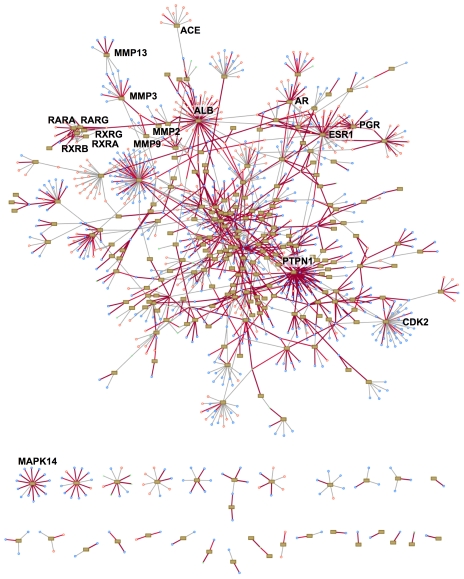
Network of known protein-drug interactions. Proteins are shown as rectangular boxes (nodes), drugs are shown as pink (approved) and blue (experimental) circles, and edges represent known interactions annotated by DrugBank. Edges colored red denote known interactions that were docked with a good icm-score. Here we show only the 252 proteins for which at least one known drug docked well – the ‘reliable-for-docking’ set. The proteins at the bottom of the graph are not connected to other proteins through shared binding drugs.

We gathered the known protein-drug interactions into a network (Figure 3) with proteins as rectangular nodes, drugs as circular nodes, and interactions as edges. Interaction edges with good docking scores were highlighted in red. Proteins from the same family were often grouped close together and shared many drug interactions, such as the retinoid X and retinoic acid receptors and the matrix metalloproteinases. Proteins having the most known drug interactions in the network included the transport proteins serum albumin and the phosphatase PTPN1. The most highly-connected chemicals in the network were metabolites: ATP, NAD, and NADP. For some proteins such as MAPK14, 13 of 14 known inhibitors were well predicted by docking, whereas for others such as ACE, only one of its nine known inhibitors scored well. For 426 of the 678 protein targets not included in Figure 3, none of their known interacting drugs could be docked well, reflecting the limitations of current molecular docking methods. To this end, we chose the subset of 252 protein targets for which at least one known drug docked well (from the 1116 interactions that docked well), deemed as more ‘reliable-for-docking’ compared to the other proteins.

### Large scale cross-docking & score thresholds

We proceeded to dock the 252 reliable protein set against the database of 4621 drugs. Considering the multiple crystal structures per protein and the multiple binding pockets per structure, there were a total of 1514 crystal structures and 2923 binding pockets. Each drug was docked to all binding pockets of a protein and whichever pocket gave the best docking score for the drug determined the final protein-drug score. This method allowed multiple conformations of a protein to be accounted for during docking and provided a simple model of protein flexibility.

In total, we docked 1.2 million protein-drug interactions. 104,625 (0.9%) had ICM docking scores (icm-score) of −30 or better, encompassing all1116 known interactions in the reliable data set. Since the fraction of known interactions in the predicted set was so low, we assumed that the vast majority of predictions were false positives. Though we believed that novel drug-target interactions existed and were enriched within these 104,625, there was clearly a need for more stringent score thresholds.

We investigated various methods of selecting top drug-target interactions. The standard software-recommended icm-score is based on a weighted sum of various binding energy terms [13]. The pmf-score, or potential of mean force score, is a measure of the statistical probability for the drug and protein to interact with each other (for example, it examines interatomic distances and atom types of the docked interaction and compares that to existing interactions in PDB) [14]. A consensus score was developed that uses both icm- and pmf- scores and allows us to select the x% of top interactions for each protein; it is described in more detail in case studies below. We also ranked interactions in two ways. The drug-rank is the rank of this drug compared to all drugs docked to this protein (from 1–4621), and the protein-rank is the rank of this protein when the drug is docked to all proteins (from 1–252). Requiring high drug and protein ranks (i.e. a low value when the two ranks are summed together) enforces a mutual specificity criterion. We hypothesized that by choosing interactions with good scores and ranks, we would better filter out false positive predictions.

**Figure 4:**
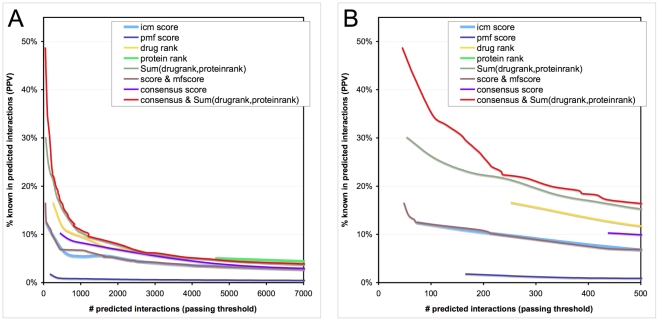
Score thresholds assessment. Various combinations of score and rank thresholds were assessed using the positive predictive value (PPV). A) shows the PPVs for thresholds predicting less than 7000 interactions. B) is a zoomed in version showing clearer PPV separation for the top 500 predicted interactions.

To assess performance, we measured the positive predictive value (PPV), defined as the proportion of predicted interactions that are known binding interactions. The premise is that a better threshold would yield a set of predictions more enriched with known interactions, and novel interactions that are more likely be true binding events. Figure 4a shows that as the stringency of a threshold increased (i.e. icm-score of −40 versus −30), fewer interactions are predicted; however, the PPV increased due to a higher proportion of known interactions in the predicted set. This behavior is consistent for all thresholds, and the highest PPVs are generally observed within the top 100 predicted interactions. It is important to note that each of the 4621 drugs will always have a top-ranked protein (interactions with protein-rank of 1), and each of the 252 proteins will always have a top-ranked drug (interactions of drug-rank 1). Thus, the protein-rank threshold particularly is not sensitive alone.

The protein-rank and pmf-score thresholds appeared to be the worst based on both the PPV plot (Figure 1) and on enrichment factors (Table 1). However, they showed better PPVs when combined with other thresholds. For example, the drug rank and protein rank measure performs much better than drug-rank alone, and the consensus score (combining icm- and pmf-score) also performs better than the icm-score alone. We measured the enrichment factor for each type of threshold, at its most stringent setting (leftmost points of Figure 4a) and found that the pmf-score and protein-rank were the least effective at predicting known drugs (Table 1). Instead, combinations of score and rank criteria provided a 100–500× enrichment of known interactions compared to a random algorithm, and a10–50× enrichment compared to a standard binding energy-based ICM score cut-off of −30. Interestingly, the drug-rank 1 and protein-rank 1 (basically the sum of ranks is 2) combination threshold performs surprisingly well; however, adding the consensus score clearly improves PPV for the top ∼300 interactions (Figure 4b) which are the most interesting to us for manual inspection.

**Table 1:** A comparison of various threshold methods based on their ability to predict a high percentage of known interactions (PPV) and enrich the predicted interaction set for known interactions.

threshold	# predicted interactions	# known in predicted interactions	# proteins in interactions	% known in predicted set (PPV)	enrichment factor versus random
random	1,164,492	1116	252	0.1%	1
icm-score of −30	104,625	1116	252	1.1%	11
pmf-score of −300	150	3	20	2.0%	21
protein-rank of 1	4621	234	206	5.1%	53
consensus score 0.05%	437	45	238	10.3%	107
icm-score of −100	72	9	17	12.5%	130
drug-rank of 1	252	42	252	16.7%	174
icm-score −100 & pmf score −140	48	8	13	16.6%	174
drug rank 1 & protein rank 1	53	16	53	30.2%	315
consensus score 0.05% & sum(drug rank, protein rank)≤4	45	22	39	48.8%	510

Thresholds are listed by increasing enrichment. It is also important to consider the size of the predicted set and how many proteins are included.

Another threshold method is to use the scores of known binders as the score cut-off for each protein. We investigated this using the best and worst icm- and pmf-scores of known drugs. Table 2 shows that this did not result in a higher enrichment, nor did it help narrow down the number of predicted interactions.

**Table 2:** A comparison of various threshold methods based on their ability to predict a high percentage of known interactions (PPV) and enrich the predicted interaction set for known interactions compared to other methods.

Threshold	# predicted interactions	# known in predicted interactions	# proteins in interactions	% known in predicted set (PPV)	enrichment factor versus random
use icm- score of worst scoring known binder	62337	1117	252	1.8%	20
use icm- & pmf- scores of worst scoring known binder	28840	716	252	2.5%	27
use icm- score of best scoring known binder	16412	253	252	1.5%	17
use icm- & pmf- scores of best scoring known binder	7859	253	252	3.2%	35

These thresholds use the best and worst scores of known binders for each protein.

Overall, the combination of consensus score with the two ranks gave the highest PPV and enrichment values: in the top 50 predicted interactions, 49% are known. This gave us confidence that many of the other 51%, all novel interactions, are real.

### Case study: MAPK14

Two examples are presented to illustrate the utility of combining rank and scoring criteria. The first is for the signaling protein MAPK14 (also known as p38 alpha), an integral component in numerous cellular processes. It is a drug-target for inflammatory diseases [17]. MAPK14 is known to be a challenging docking target due to its structural flexibility [18] and its shallow binding pocket [19]. However, these docking studies used only one 3D structure. In our dataset, there are 35 crystal structures of MAPK14 in different conformations, providing a simple view of protein flexibility.

**Figure 5:**
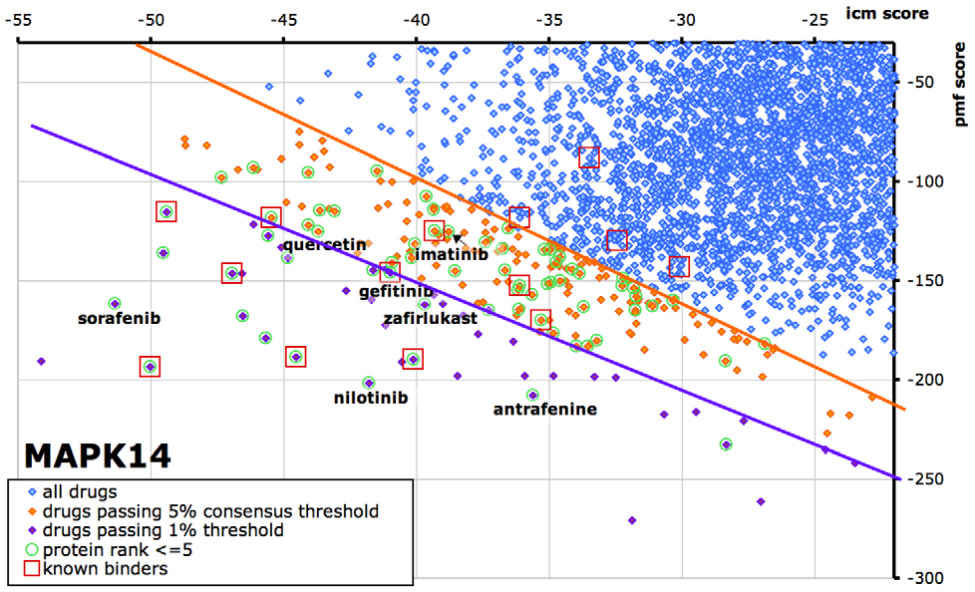
A score-plot containing docking ICM- and pmf- scores for 4621 drugs to MAPK14. Each point represents a drug. The top 5% of the drugs as determined by the consensus scoring threshold are shown as orange dots. These drugs were also docked to the 252 other drug targets in our database, and circles denote the drugs for which this protein was one of the top 5 targets for the drug. The circle colors denote whether the protein rank was based on the ICM score (green) or the pmf score (purple). Finally, drugs that are known to bind MAPK14 are shown in red boxes, and it can be seen than most of these red boxes pass both the consensus and protein rank thresholds.

The consensus score is based on the observation that when docking a large number of diverse compounds to any target, most compounds have poor icm- or pmf- scores, and few compounds have both good icm- and pmf- scores. Therefore, we chose a linear threshold that eliminated the densest area of points in the poor scoring region (top-right) of a score plot like Figure 5, and selects the compounds in the best scoring region (bottom-left) as potential interaction hits. As seen in Figure 4a and Table 1, the consensus score performed better for PPVs and enrichments compared to a simple icm- and pmf- score combination.

Figure 5 plots the icm- versus pmf- scores of the 4621 drugs docked to MAPK14. Each drug is a point on the graph, where the 5% of drugs passing a consensus threshold are shown in orange, and the 1% passing a consensus threshold are shown in purple. For 67 drugs, MAPK14 was one of the top 5 scoring targets; they are circled in green. Table 3 shows that a combination of the consensus and protein rank criteria resulted in the best enrichment (110×) of known drugs. There were 15 annotated known binders of MAPK14 in DrugBank, but we disregarded 2-chlorophenyl due to it being a very small molecule with a very weak MAPK14-binding affinity (>1 mM). 10 of 14 known drugs were predicted through our stringent thresholds. Though 4 true positive binders were lost, 99.99% of points were eliminated, presumably consisting mostly of non-binders. Through literature search, we found that imatinib and quercetin have been previously tested against MAPK14 and did not show any inhibition [20]. This suggested that the 5% consensus threshold was too lenient for MAPK14, whereas the 1% was more appropriate. Within the other approved drugs predicted to bind MAPK14, we found literature validation for sorafenib, a multi-kinase inhibitor approved for renal cell carcinoma [21], and gefitinib, a EGFR inhibitor approved for late stage non-small cell lung cancer [22].

**Table 3:** Enrichment factors of various thresholds for MAPK14.

	all docked drugs	known drugs ligands	enrichment factor versus random
# docked to MAPK14	4621	14	1
# passing icm score ≤−30	970	14	5
# passing 5% consensus score	225	10	15
# passing 5% consensus & protein rank ≤5	67	10	49
# passing 1% consensus score	45	6	44
# passing 1% consensus & protein rank ≤5	18	6	110

**Figure 6:**
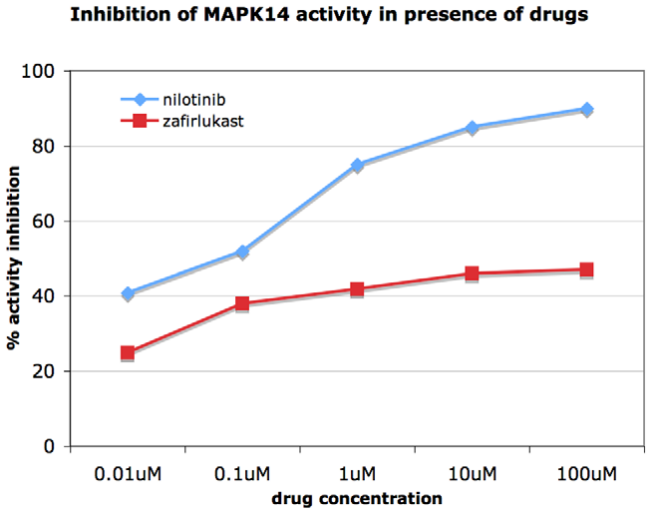
Testing nilotinib and zafirlukast in ATP-competitive enzymatic assays against MAPK14. Results are plotted as percent inhibition of activity versus drug concentration. The nilotinib-MAPK14 IC50 was calculated to be 40 nM.

Previous high-throughput studies have shown varying results regarding nilotinib-MAPK14 inhibition. Some enzymatic assays to MAPK14 showed weak inhibition: 570 nM or 2.2 µM depending on the assay type [23]. Direct binding assays have shown 100 nM Kd [23] or no binding at all in peptide pulldown experiment [20]. Since nilotinib was one of our top approved drugs predicted to bind MAPK14, we decided to further experimentally validate the interaction. We performed MAPK14 ATP-competitive binding assays for two inhibitors that were available for purchase: zafirlukast, and nilotinib. As seen in Figure 6, both drugs exhibited inhibition of MAPK14 at therapeutically relevant concentrations (<10 µM) in a dose dependent manner. Zafirlukast (AstraZeneca) is an oral leukotriene inhibitor that reduces inflammation of breathing passage in asthma patients. We found that it does inhibit MAPK14 weakly, and this may contribute to its inflammation reducing effect. The chronic myeloid leukemia drug nilotinib was especially potent with an IC50 of 40 nM.

Despite their appeal as an inflammatory disease target, MAPK14 drug candidates to date have failed due to drug toxicity issues [24]. Though it may seem underwhelming to use a cancer drug with potentially serious side effects to treat inflammation, nilotinib is noted to have a much milder adverse effects profile compared to its similar drug dasatinib [20]. Another similar drug imatinib has shown promise in treating rheumatoid arthritis in mouse models [25] and specific patients [26], [27], speculated due to its inhibition of mast cell c-Kit and PDGFRB. Nilotinib also inhibits these two proteins, and its extra inhibition of MAPK14 may render it a better choice for arthritis models. Recently, nilotinib was tested in a glucose-6-phosphate-isomerase-induced arthritis mouse model and found to significantly prevent paw inflammation – to a greater extent than imatinib [28]. This study also suggested that the two drugs acted through some distinct mechanisms. Overall, these findings seem to agree with our observations that nilotinib potently inhibits MAPK14, unlike imatinib, and thus has added potential as an anti-inflammatory drug.

### Case study: BIM-8

**Figure 7:**
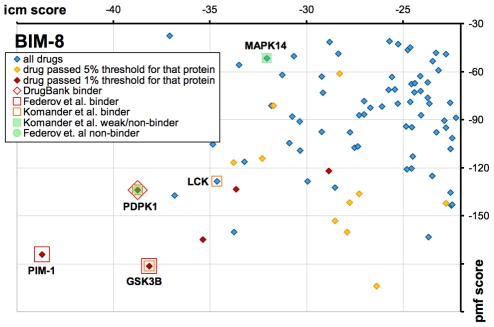
Docking icm- and pmf- scores for BIM-8 docked to 252 reliable-for-docking protein targets. Each point represents a protein target. Targets for which BIM-8 passed a consensus threshold are shown as orange dots (top 5%) and brown dots (top 1%). Targets with experimental support are enclosed in red colors. Targets that have shown no binding activity with BIM-8 in the literature are shown in shades of green. It can be seen that most of the actual targets of BIM-8 pass stringent consensus score thresholds.

A second example is the Protein Kinase C inhibitor BIM-8. We docked BIM-8 to the set of 252 reliable targets, and the results are plotted in Figure 7. Each point on the graph represents a protein target, and targets for which BIM-8 passes the 5% consensus threshold are shown in orange.

We compared our results to three previous studies. Two studies performed protein kinase assays with radioactive ATP and substrate peptides, where inhibitor binding decreases the amount of radioactive peptide produced [29], [30]. The third study performed thermal shift assays where inhibitor binding increased the kinase stability and thus the melting point [31]. BIM-8 targets discovered by these papers are shown in shades of red in Figure 7, and non-binders in these papers are shown green. The only annotated target of BIM-8 in DrugBank is PDPK1. GSK3B and PIM1, which are in the top 5 protein rank and top 5% consensus threshold, were also validated as inhibitors. PDPK1 was not found to be an inhibitor by the first two studies but was confirmed as a binder by the third study with a kinase assay and crystal structure. Overall, if we count that there are 4 known binders (PIM1, PDPK1, GSK3B, LCK, since CDK and MAPK14 are probably weak or nonbinders), we can see that applying a 5% consensus threshold and protein rank criteria gave us a 63-fold enrichment over random selection, and a 63/10.5 = 46 fold enrichment over using a standard ICM score threshold of −30 (Table 4).

**Table 4:** Enrichment factors of various thresholds for BIM-8.

	all docked proteins	known protein targets	enrichment factor versus random
# proteins BIM-8 was docked to	252	4	1.0
# passing default score ≤−30	24	4	10.5
# passing 5% consensus score	20	4	12.6
# passing 1% consensus score	6	3	31.5
# passing 5% consensus & protein rank ≤5	3	3	63
# passing 1% consensus & protein rank ≤5	3	3	63

### Drug-target interaction map

**Figure 8:**
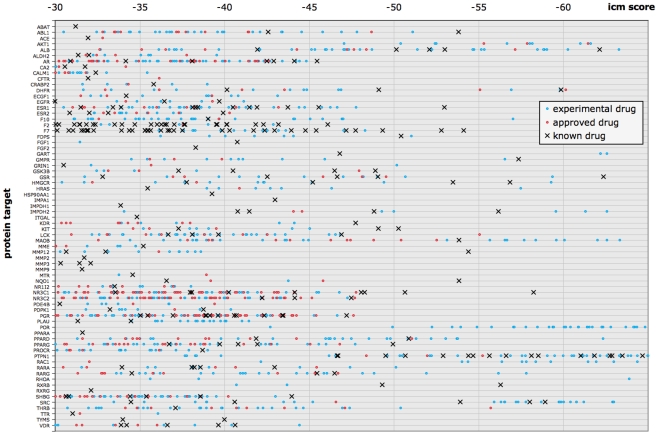
Quantitative interaction map of drugs docked to protein targets, according to their ICM docking score. Each protein is represented by a column, on which a black cross denotes a known drug docked to the target, a red dot denotes an approved drug docked to the target, and a blue dot denotes an experimental drug docked to the target. Only the top predictions for established drug targets (at least one known approved drug) that docked with a score passing the consensus threshold and had a protein-rank ≤5 are shown.

For a global and quantitative review of the predicted protein-drug interactions, we plotted the icm scores of drugs docked to established drug targets (Figure 8). Each protein is represented by a column, on which a black line denotes a known drug docked to the target, a red dot denotes an approved drug docked to the target, and a blue dot denotes an experimental drug docked to the target. Only protein-drug interactions that docked with a score passing the consensus threshold and had a protein-rank ≤5 are shown.

Overall, the known drugs (black crosses) had better scores than other drugs for a given target. This was expected, as many of these known drugs were chemically optimized for their targets. For a number of targets, the known drug was the only predicted interaction. None of the approved and experimental drugs from DrugBank were able to dock well, despite a reliable protein structure, suggesting that virtually screening larger chemical databases may be the only way to discover novel inhibitors by docking. For most targets, at least one experimental drug showed a better score than the known drugs; however, experimental drugs are often unavailable for purchase or experimental testing. Instead, we were most interested in cases with approved drugs such as the MAPK14-sorafenib example which was verified by the literature, and the MAPK14-nilotinib example which we verified with an in vitro kinase assay.

Through literature search, we found experimental support for many of our top drug-target predictions that scored better than known interactions (Table 5). These all pass the 1% consensus threshold and are observed to have high drug and protein ranks for the most part. It is important to note that the drug-rank depends on the number of known binders for the protein; thus, since ESR1 had 39 annotated drugs in DrugBank, a drug-rank of 16 is not as low. In contrast, a drug-rank of 16 would be low for MMP13, which has only seven annotated drugs in DrugBank.

**Table 5:** Top predicted hits that have literature support.

protein	drug	icm score	pmf score	drug rank	protein rank	notes
AIFM1	DB02332	−79	−231	1	1	Flavin is a cofactor. [51]
ALB	DB03756	−66	−163	1	2	Dosahexanoic acid (DHA) can form complex with albumin and confers neuroprotective effects in rats. [40]
ALB	DB06689	−51	−130	84	3	Ethanolamine oleate promptly binds with albumin in the blood [52]
AKT1	DB03265	−81	−95	2	1	Crystal structure of inositol 1,3,4,5-tetrakisphosphate bound to AKT1. [53]
BTK	DB03344	−69	−99	1	3	[54] shows that inositol 1,3,4,5-tetrakisphosphate binds to BTK. This compound is very similar: inositol 1,3,4,5,6 tetrakisphosphate.
CYB5R3	DB02332	−71	−258	2	2	Flavin is a cofactor. [55]
ESR1	DB05414	−47	−197	3	1	ERA-923 is a selective estrogen receptor modulator. [32]
ESR1	DB01645	−42	−109	16	1	Genistein is a selective estrogen receptor modulator. [33]
GART	DB02223	−63	−126	1	5	LY-231514 tetra-glu a known thymidylate synthase inhibitor. LY-231514 is pemetrexed, a GART and thymidylate sythase inhibitor. inhibitor. [56]
GART	DB02794	−62	−147	2	4	Crystal structure of compound bound to E.coli GART. [57]
GSR	DB02332	−57	−211			Flavin is a cofactor. [61]
KDR	DB04879	−49	−152	1	1	Vatalanib is a pan VEGFR inhibitor. IC50 37 nM. [37]
KIT	DB04868	−44	−240	4	2	Nilotinib. [38]
MAPK10	DB00317	−39	−183	72	3	Gefitinib binds MAPK10 weakly: Kd = 2–3 uM. [58]
MAPK14	DB00398	−51	−161	2	2	Sorafenib IC50 0.057 uM. [59]
MMP2	DB02255	−37	−84	1	6	Illomastat is a broad-spectrum MMP inhibitor. Ki 0.5 nM (Chemicon International Inc, Temecula, CA)
MMP8	DB02255	−44	−67	2	1	Illomastat is a broad-spectrum MMP inhibitor. Ki 0.1 nM (Chemicon International Inc, Temecula, CA)
NR3C2	DB01395	−48	−150	1	1	Drospirenone, a progestogen with antimineralocorticoid properties. [60]
PPARD	DB03756	−62	−144	1	4	DHA can activate PPARD. [61]
PPARG	DB06536	−47	−130	9	1	Tesaglitazir is a dual PPARA/PPARG agonist [62]
RAC1	DB03532	−120	−145	1	1	RAC1 is a GTPase [51], and this compound is a standard GTP analog.
RARG	DB02466	−58	−216	1	1	BMS181156 binds RARG with Kd 0.6 nM. [63]
RARG	DB02258	−56	−220	2	1	SR11254 is a RARG-selective ligand [64].
RARA	DB05076	−45	−131	6	2	4-HPR is a highly selective activator of retinoid receptors. [65]
RARG	DB05076	−46	−134	6	1	4-HPR is a highly selective activator of retinoid receptors. [65]
RARG	DB02741	−52	−217	3	1	CD564 binds RARG with Kd 3 nM. [63]
RARG	DB03466	−46	−208	11	1	BMS184394. [63]
RXRA	DB03756	−54	−137	1	8	DHA. [66]
RXRA	DB04557	−53	−156	2	5	Arachidonic acid. lit support. [63]
VDR	DB04891	−49	−204	1	1	Becocalcidiol, a vitamin D analog. [34]
VDR	DB04295	−44	−297	4	1	ED-71, a vitamin D analog. [35]

One type of validated interaction includes drugs that are close analogs of known drugs for that target; for example, the estrogen analog ERA-923 is a known selective estrogen receptor modular (SERM) [32]. Genistein is known to bind both ESR1 and ESR2 [33]. Becocalcidol and ED-71 are vitamin-D analogs and bind the vitamin D receptor [34], [35]. Drosiprenone is a synthetic progestin with anti-mineralocorticoid receptor (MR, NR3C2) effects and has potential for reducing cardiovascular risk in women taking oral contraceptives or postmenopausal hormone treatment [36]. Due to the many in depth studies on kinase inhibitor specificity, we were able to find collaborating evidence for some of our kinase protein interaction predictions. For example, vatalanib is a known pan-VEGFR inhibitor [37], nilotinib is a potent KIT inhibitor [38], and other inhibitors of MAPK14 and targets of kinase inhibitor BIM-8 were discussed in previous sections. Docosahexanoic acid (DHA, DB03756) is an endogenous ligand for brain fatty acid binding protein (B-FABP) that is essential for brain growth and function [39]. We predicted that it binds the transport protein human serum albumin; indeed, this interaction has been validated and found to confer neuroprotection in animal models of ischemia [40]. This finding suggested that DHA might have potential repositioning value for ischemic stroke.

Overall, we were able to find literature support for 30 of our top predicted interactions, which validated our computational method as useful for finding novel drug-target interactions.

## Discussion

The binding of a small molecule drug to its target protein in a cell is much more complex than a single docking calculation. For example, an ATP-competitive kinase drug would have hundreds of ATP-binding sites to choose from due to the large size of the kinome. Cancer drugs such as sunitinib are now known to potently inhibit many more kinase targets than previously expected [41]. In addition, non-kinase targets of kinase drugs have also been found: NQO2 was the first non-kinase target discovered for imatinib [20], [42], and several cytotoxic LIM kinase inhibitors were found to be actually inhibiting tubulin [43]. Such studies imply that the target search space for any inhibitor should be the entire druggable proteome.

Our strategy was to find novel drug targets of existing drugs by computationally screening the druggable proteome. For this purpose, we chose molecular docking due to its speed, low cost, and detailed three-dimensional simulation. Moreover, docking can evaluate any protein with a solved structure due to its virtual nature, without the need for tailoring enzymatic assays or collecting drugs in solutions. However, docking is known to have a high false positive prediction rate, due to limitations such as incomplete binding pocket prediction, inadequate ligand conformation sampling, inaccurate scoring functions, lack of protein flexibility, and lack of water and cofactor molecules during the simulation. As evidenced in this study, only 31% of the 3570 known interactions docked with a good score. One review states that 10–50% of a set of diverse compounds can be expected to be docked correctly for a given target [12]. We are well within this range, and believe our method performs quite well considering the large variety protein targets involved and the automated nature of the pipeline. However, the other 69% of known interactions were not predicted due to docking limitations.

Our method attempted to address these limitations. First, we manually included binding pockets that were present in PDB structure complexes but not predicted by the binding pocket search. Second, we docked each interaction 10 times to better sample ligand conformations. Third, we applied consensus score and rank criteria to further narrow down top scoring docking hits. Fourth, we used all available structures of a protein (versus choosing one representative structure), to allow a simple view of protein flexibility. We did not incorporate water and cofactor molecules in our docking simulations due to the computational complexity involved. However, by selecting proteins for which at least one known drug docked and scored well, we selected proteins for which the limitations of the docking software were not critical for a good prediction. In short, assuming the docked conformation of the known ligand was correct, we used only proteins for which the binding pocket was genuine, the scoring functions were adequate, the protein was in a conformation amenable for drug inhibition, and the lack of water or cofactor molecules didn't drastically affect the prediction.

Virtual screening studies typically involve docking large chemical databases to one protein target, selecting compounds that score within the top 0.5–1% of the database and then further prioritizing them by visual examination. When experimentally validating these top candidates, a 5% hit rate can be considered a successful endeavor (where a good hit is a predicted compound showing an experimental binding affinity in the µM or lower range) [44]. Depending on the target, the crystal structure, the software used, post-docking criteria (such as chemical clustering), and even the individual performing the visual examination, the hit rate can be improved to 10–40% (Cavasotto *et al.* had 14% hit rate from 50 tested compounds [15]; Sabio *et al.* had a 36% hit rate from 56 tested compounds [45]).

In our case, both the standard scoring threshold and the known-inhibitor score were not sufficient. With a normal score threshold of −30, docking 4621 drugs against 252 proteins resulted in 104,625 predicted interactions. This is roughly 1% of the docked interactions, so even selecting the top 1% of the docking hits for validation becomes prohibitive for large-scale studies. It is important to note that each protein has different physiochemical properties: for some proteins, hundreds of compounds pass the −30 cut-off, while for other proteins none pass. Thus, using the known-inhibitor score as a cut-off allows for a threshold that is tailored to each protein. However, this method still predicted ∼8000 interactions at the most stringent. Our consensus threshold allowed us to pick the top 1% (or any x%) of docked compounds with the best icm- and pmf- scores for each protein and further filter from there. Through testing many combinations, we found that using the consensus score with rank information allowed us the highest PPV – nearly 50% - and enrichment factor – 50 times better than standard −30 score threshold and 490 times better than random selection. This high enrichment for known interactions suggests that many of the other predictions that have not yet been experimentally tested may be true binding interactions.

There are limitations to this scoring scheme. Since the pmf-score is a statistical score comparing the docked interaction to known interactions in PDB, a chemical with a different scaffold or novel binding conformation may have a poor pmf-score and become predicted as a false negative. However, our foremost goal in this study was to eliminate as many false positive predictions as possible and obtain a high enrichment of true positives in our predicted interaction set. Thus, it was acceptable to miss some false negative predictions. In addition, the consensus score is quite simple with a linear separation method, and may not be as informative as a machine-learning algorithm that trains on known ligand docking scores. However, we desired an automated scoring method that did not depend upon the existence of known ligands. That is, if a protein structure had just one, or no known binders, our method would still be able to select the top 1% of docking hits.

To date, cross-docking of proteins to compounds has generally been used for small datasets. As an example, Huang *et al.* docked 40 targets against 40 compounds to check whether their docking method could distinguish between a target's cognate ligands and the other targets' cognate ligands [19]. In this large-scale cross-docking study, our use of a 1000-processor cluster was essential to completing the tens of millions of docking simulations in a timely manner. In addition, the large number of crystal structures and binding pockets involved required much of the docking pipeline be automated.

High-throughput computational screening of drug-target interactions represents a parallel approach to high-throughput experimental screening. Due to differences in experimental methods, assay settings, and protein panels, different studies may present differing results. For example, small molecule affinity purification methods that use whole cell lysates would give different results from *in vitro* kinase assays that use a specific panel of proteins. In the case of gefitinib, two such studies had distinct differences in their proposed cellular targets [22], [41]. Differences in methods are also further compared in a study by Manley et al [23]. We presented an example for BIM-8, which binds to PDPK1 differently in two similar *in vitro* experiments. For MAPK14, the experimental results for nilotinib also varied. We experimentally tested two purchasable approved drugs against MAPK14 and found that nilotinib was a strong nanomolar inhibitor, and zafirlukast was also an inhibitor, though not as potent. Thus, interactions that are predicted to be very likely inhibitors computationally may merit extra study even if experimental tests are initially negative.

In short, we have developed a computational pipeline that can run large-scale cross-docking of compounds to targets. We developed stringent criteria to filter a large proportion of false positive interactions. The two case studies presented were selected based on known experimental binding assay data, so as to demonstrate the notable enrichment of known interactions using our scoring and ranking criteria. We hypothesized that predicting a set of interactions with a higher PPV (enrichment of known interactions) would also lend confidence to the other novel interactions in the set. This appears to have worked, as we were able to find validation for 31 predicted drug-target interactions that were not previously annotated in DrugBank, as well as validate two other inhibitors of MAPK14. Other drug-target interaction predictions are currently undergoing experimental validation; novel interactions discovered are potential drug repositioning candidates, but also provide insight into a drug's mechanism of action and adverse effects profile.

## Methods

### Pocket database and drug database construction

We downloaded the DrugBank 2.5 database [46], containing drug information and comprehensive information of their targets. We extracted human protein drug targets from DrugBank and retrieved their sequences from SwissProt [47]. Protein Data Bank structures showing at least 95% sequence identity for proteins at least 20 amino acids in size were downloaded. They were required to be X-ray crystal structures with a minimum resolution of 2.8 Å. Multiple chains were grouped into a set of non-redundant sequences, based on PDB's chain redundancy analysis at the 95% sequence identity level.

### Preparing a target pocket database

We prepared protein structures for docking using Molsoft's ICM software version 3.4-9c [13], removing water molecules, solvent ions, and other ligands from the structures. We added hydrogen atoms to the structures then optimized their positions. These prepared protein structure files can be downloaded from http://www.bcgsc.ca/downloads/yli/. To predict pockets, or potential binding sites, we used the PocketFinder [48] method in ICM, which calculates a transformation of the van der Waals energy for an aliphatic carbon probe on a grid map. For each protein, the three largest pockets are retained in the database. If metal ions were found near a pocket, we prepared two receptors for docking, one of the protein with the metal ion and one without.

The receptor was defined as the box 3.5 Å surrounding the pocket. If the pocket overlapped well with the ligand but the ligand extended out of the protein structure, we defined the receptor be the box 3.5 Å around the pocket but also including 2.0 Å around the ligand. This ensured that known ligand binding sites not predicted by our automated method were also included in our pocket database.

### Docking

We docked drugs to target receptors using the ICM virtual library screening (VLS) module. This method performs rigid-receptor flexible-ligand docking using a two-step Monte Carlo minimization method and energy scoring function to sample ligand conformations and select the best docking hits. MMFF partial charges and ECEPP/3 force-field parameters are used. Docking one interaction required on average 30 seconds to 1 min per processor. A given protein may have several structures, each of which with more than one pocket; in such cases we dock all pockets to a drug, and the best scoring interaction is selected to be the representative protein-drug score.

To ensure a sufficient coverage of the docking energy landscape, we docked each drug-target interaction 10 times in the known docking analysis and 5 times in the large-scale cross-docking analysis. Docking was performed on a Linux cluster with 1000 processors – this level of throughput allowed us to complete 1–3 million dockings per day.

### Known interactions docking

8867 known interactions between human protein targets and drugs were culled from the DrugBank Drugcards database. Of these, 3570 interactions with protein target crystal structures present in our database were docked. Due to the Monte-Carlo nature of the ICM method, each interaction was docked 10 times to better cover the docking energy landscape. After 10 iterations, the best scoring prediction was retained.

If the protein structure was solved in complex with a ligand, a Tanimoto coefficient was used to determine if the docked drug was similar to the complexed ligand. A coefficient less than 0.54 represented similar molecules [49], and thus cognate dockings. Evaluation of static RMSD values of protein-drug interactions representing 380 cognate interaction dockings was performed on a case-by-case basis as the chemical numbering of PDB heteroatoms and docked structures often differed, which caused incorrect RMSD calculations. Each RMSD comparison was required to match at least 30% of the docked ligand atoms to the cognate crystal-structure ligand. 320 interactions pass this requirement, of which 221 (69%) showed RMSDs under 2 Å. The other 99 (31%) had RMSDs larger than 2 Å.

Cytoscape [50] was used to generate the known drug-target interaction map. Networks were fitted to a force-directed layout and manually edited for improved visibility. Drugs and protein targets are nodes in the network, interconnected by interaction edges. The edge lengths were not weighted, and are adjusted for maximum visible understanding.

### Applying and evaluating score thresholds

We applied several methods of score thresholding: applying cut-offs of the ICM docking score ranging from [−25 to −100]; applying cut-offs of the ICM potential of mean force score ranging from [−80 to −200]; applying a drug rank cut-off ranging from [1 to 4500]; applying a protein rank cut-off ranging from [1 to 252]; applying a combined docking score and mean force score cut-offs. For the consensus score thresholds, all slopes (from −1 to −40) and intercept (from 0 to −400) combinations were tested. For each line, we calculated the density of the points eliminated in a trapezoidal area delineated by the consensus line, the best icm- score for this protein, and the best pmf-score for this protein, the midpoint between the worst icm-score and its mean, and the midpoint between the worst pmf-score and its mean. For two consensus thresholds that predicted the same number of interactions, we used the one that eliminated a denser cloud of points.

While evaluating PPV for combination thresholds, it was often observed that two sets of thresholds resulted in the same number of predicted interactions but different PPVs. In such cases, we considered only the threshold combination that gave us the higher PPV.

### Large scale cross-docking

1,164,492 interactions between 252 proteins and 4621 drugs were docked using ICM. Though there were actually 4854 drugs small molecules, some were excluded being too small or too large for docking (molecular weight under 100 or over 1000 g/mol). Due to the multiple binding pockets per protein and multiple crystal structures per protein, there were a total of 2923 binding pockets. Each interaction was docked 5 times to better cover the docking energy landscape and the best scoring conformation was retained. Overall there were 2923×4621×5 dockings or 68 million docking calculations. The icm and pmf scores of each interaction were gathered into large matrices for further analysis.

### Kinase assays

Protein inhibition assays were performed by SignalChem (Richmond, BC, Canada). Kinases assays consisted of ^33^P-ATP at 25 µM, the protein kinase, peptide substrate, assay buffer, and the drug. Blank assays without substrate or drug, and assays without the drug, were used as controls. Staurosporine at 1 µM was used as the positive control drug.
